# Healthcare experiences of patients with age-related macular degeneration: have things improved? Cross-sectional survey responses of Macular Society members in 2013 compared with 1999

**DOI:** 10.1136/bmjopen-2016-012790

**Published:** 2017-02-06

**Authors:** Emily M Boxell, Winfried M Amoaku, Clare Bradley

**Affiliations:** 1 Health Psychology Research Unit, Royal Holloway, University of London, Egham, UK; 2 Department of Psychology, Royal Holloway, University of London, Egham, UK; 3 Ophthalmology, DCN, University of Nottingham, B Floor Eye and ENT Centre, University Hospital, QMC, Nottingham, UK; 4 Health Psychology Research Ltd, Royal Holloway, University of London, Egham, UK

**Keywords:** age-related macular degeneration, patient satisfaction, sight impairment, information provision

## Abstract

**Objective:**

To investigate healthcare experiences of patients with age-related macular degeneration (AMD) and determine whether a previous survey and Royal College of Ophthalmologists (RCOphth) management guidelines brought improvements.

**Design:**

Cross-sectional survey of Macular Society members in 2013 compared with previous 1999 survey.

**Setting:**

UK Postal Questionnaires.

**Participants:**

1169 respondents in 2013 (1187 in 1999).

**Intervention:**

Publication of 1999 survey results (2002), and RCOphth AMD guidelines (2009).

**Main outcome measures:**

Respondents answered questions about experiences at diagnosis. Five questions were replicated from the 1999 survey for direct comparison in the 2013 survey which included additional questions based on 2009 RCOphth recommendations for information and support provision for patients with AMD.

**Results:**

Most 2013 survey respondents were given the name of their macular condition (91%), felt the healthcare professional was interested in them (71%) and were satisfied overall with the diagnostic consultation (76%). These outcomes show significant improvement since 1999. Within the 2013 sample, multivariable analyses showed gradual trends of improvement over time in: provision of written information, Macular Society information and receiving appropriate help, support and advice at diagnosis. Only overall satisfaction with the diagnostic consultation (but not the other nine areas of information and support provision studied) significantly improved in the time after publication of the RCOphth 2009 guidelines. There were no significant improvements associated with the publication of the 1999 survey results. Low information and support provision remained, for example, 44% of respondents diagnosed after the RCOphth 2009 guidelines reported not receiving information on what to do if vision deteriorated. Lack of such information at diagnosis was significantly associated with registration as sight impaired (p<0.01). Reports of general practitioner (GP) knowledge of AMD remained low: 39% reported their GP was ‘not at all well informed’. The 2013 respondents reported lower levels of help and support from GPs than 1999 respondents (p<0.001).

**Conclusions:**

Patients diagnosed with AMD after 1999 (vs before 1999) reported better experiences at diagnostic consultation. However, information and support provision at diagnosis, and satisfaction with GPs remained low.

**Strengths and limitations of this study**

- This is the first large-scale, nationwide survey to examine whether there were changes in healthcare satisfaction following interventions designed to improve experiences of patients with age-related macular degeneration. It is also unique in examining whether respondents felt they were receiving appropriate support for AMD from their general practitioners.
- The results are timely given that there are increasing numbers of people diagnosed with AMD in line with an ageing population.
- The main analysis adjusted for differences in sociodemographic and eye-related factors (eg, registration status).
- Respondents were members of the Macular Society whose healthcare experiences may not be representative of the general AMD population.
- The survey asked respondents to reflect on their experiences at the time of diagnosis. It is possible that recall bias could affect responses. Literature on autobiographical memory suggests that women recall more details of events than men. In this study, however, men were more likely to report receiving several aspects of information and support provision than women. This suggests that difficulties with recall were not the main problem here, but rather women were less likely to be given information and support than men.

## Introduction

Age-related macular degeneration (AMD) is a progressive chronic eye condition affecting people aged 50 years and above.^1^ AMD may be asymptomatic in the early stages, but results in vision loss in the late stages of geographic atrophy (dry AMD) or neovascular (wet) AMD. Dry AMD can convert to, or be associated with, wet AMD in the same or contralateral eye. AMD is the leading cause of blindness in developed countries.^2^ There were ∼513 000 people living with late AMD in the UK in 2012, and numbers are expected to grow by a third by 2020 with the increasing age of the population.^3^ There are no proven treatments for dry AMD to date although progression may be reduced with the Age-Related Eye Disease Study 2 (AREDS 2)formula nutritional supplements.^4^ ^5^ Significant advances have been made in treatment for wet AMD with intravitreal injections of anti-VEGF drugs. Ranibizumab (Lucentis, Genentech/Novartis) was approved for use in the UK by the National Institute for Health and Care Excellence (NICE) in August 2008,^6^ and has been successful in preventing vision loss.^7–10^ More recently, aflibercept (Eylea, Regeneron/Bayer) was approved by NICE in July 2013.^11^ In addition, the off-license use of bevacizumab (Avastin, Roche/Genentech) has been advocated by some specialists for the same indication since 2006^12^ and has been found to have similar efficacy to ranibizumab.^13^

In 1999, a nationwide survey was sent to members of the Macular Disease Society (now the Macular Society), a British charitable organisation supporting people with a macular condition. The results of the survey appeared in the *British Journal of Ophthalmology.*^14^ The Macular Disease Society Questionnaire (henceforth referred to as ‘MDSQ 1999’) used in the survey was designed in response to reports of unsatisfactory healthcare experiences from members of a local group of the Society. Key findings from the survey included: over 50% of respondents thought that the eye specialist who first diagnosed their macular condition was not interested in them as a person, and 41% reported being dissatisfied with the diagnostic consultation. Respondents were asked to give their reasons for dissatisfaction. The most common reason was the attitude of the eye specialists; they were commonly seen as being dismissive, patronising, brusque or unfeeling. The second most cited reason for dissatisfaction was the lack of information provision to patients about their condition and/ or what further help was available. Experiences with general practitioners (GPs) were not much better. Twice as many respondents reported that their GP was ‘not at all well informed’ about their macular condition compared with those who said their GP was ‘very well informed’. About equal numbers reported that their GP was ‘very helpful and supportive’ about their macular condition, or ‘not at all helpful and supportive’.

The Royal College of Ophthalmologists (RCOphth) subsequently produced guidelines for AMD. Initial guidance focused on possible treatment options^12^ ^15^ but guidelines first published in February 2009^1^ (updated September 2013^16^) set standards for best practice and included recommendations about information to be communicated to patients within the diagnostic consultation. The guidelines stated that all patients require: a clear diagnosis (ensuring patients know the name of their condition), the prognosis and what to do if vision deteriorates, written information for patient and relatives and signposting to other organisations such as the Macular Society for further help and support. The guidelines also highlighted the importance of an awareness of the impact of a diagnosis of this progressive eye condition, and the need to show empathy with patients. Moreover, the guidelines emphasised that patients require information about the possibility of experiencing visual hallucinations (Charles Bonnet Syndrome (CBS)) in order to avoid distress resulting from incorrectly attributing the cause of these hallucinations, for example, to dementia.

A second large-scale nationwide survey was funded by the Macular Society in 2013 in order to establish if healthcare experiences had changed for people diagnosed with AMD since the 1999 survey. We investigated whether significant improvements had been made since publication of the 1999 survey results in 2002, and/or the RCOphth AMD guidelines in 2009.

## Methods

### Participants

**Figure 1:**
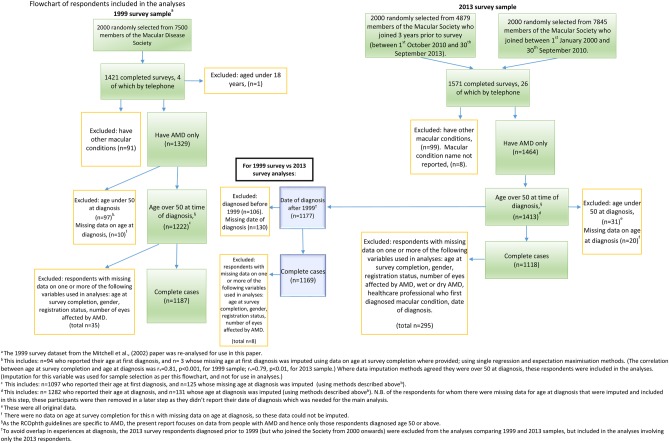
Flow chart of respondents included in the analyses.

A total of 4000 members of the Macular Society who joined after 1 January 2000 were selected from the membership database using campaign management software (NFP CARE, Advance Computer Software Group). In order to achieve an adequate sample size to investigate the impact of the RCOphth guidelines, we stratified the sampling, based on date of joining the Macular Society (as a proxy for date of diagnosis). Two thousand of the total 4000 members sampled were randomly selected from a total sample of 4879 members who had joined within 3 years prior to the 2013 survey being undertaken (ie, between 1 October 2010 and 30 September 2013) (referred to as ‘recently joined’). A further 2000 members were randomly selected from the 7845 members who had joined the Society between 1 January 2000 and 30 September 2010. Further selection criteria and the number of respondents included/excluded are summarised in figure 1.

The MDSQ 1999 (following pilot testing and clarification as required) was sent to 2000 randomly selected members of the Macular Society in 1999 and 1421 completed surveys were received (71% response rate). The Macular Society has since sent other surveys to its members, and response rates have fallen (eg, Cox and ffytche, 2014,^17^ obtained a response rate of 31%, n=1254). A total of four thousand 2013 surveys were mailed in order to achieve a comparable sample size with the MDSQ 1999.

### Materials

Three key questions on experiences in the diagnostic consultation from the MDSQ 1999 were replicated to enable comparison over time. Seven new questions were designed to assess the incorporation of the RCOphth guidelines into practice, and focus on information and support provision around the time of diagnosis. Sociodemographic information (age, gender) was collected. Eye-related information (wet or dry AMD, registration status, AMD in one or both eyes and date of first diagnosis) was also gathered together with information about which healthcare professional (HCP) the respondent considered as the first to diagnose their macular condition. The wording of the questions replicated from the 1999 survey was modified as needed to allow for the more recent development whereby optometrists are now allowed to diagnose AMD whereas in 1999 only ophthalmologists were entitled to give people a diagnosis of AMD.

Two questions on experiences with GPs were replicated from the 1999 survey. Responses were made on a Likert Scale of 0 (not at all) to 3 (very). In the 1999 survey, missing data on responses to the two GP questions were considerable. Many wrote on the survey that they had not seen their GP about their macular condition.^14^ A ‘not applicable’ option was therefore added in the 2013 survey to investigate how many had not visited their GP about their AMD.

Similar to the MDSQ 1999, the Macular Society Questionnaire 2013 (MSQ 2013) was designed for self-completion by people with a macular condition and pilot tested.^14^ Telephone interviews were offered for both surveys if needed.

### Procedure

The Macular Society sent the postal surveys to members in November 2013. Both 1999 and 2013 surveys provided free-post return envelopes. Adverts informing members that the surveys would be sent to randomly selected members of the Macular Society appeared in SideView (the membership magazine). No reminders were sent out to maintain members’ confidentiality.

Twenty six respondents requested telephone survey completion: all were conducted by EB.

### Analysis

To explore the impact of the RCOphth guidelines published in February 2009, and the publication of the MDSQ 1999 results in July 2002, a new variable was created based on the 2013 survey respondents’ date of diagnosis (before or after each of these dates). We explored sociodemographic differences between these three groups within the 2013 survey data and between the 1999 and 2013 samples, using Pearson χ^2^ analyses, one-way independent ANOVA'S or *t*-tests (or the non-parametric equivalent, Mann-Whitney test, where required). Significant results were followed up with post hoc evaluation of results (eg, examining adjusted residuals for χ^2^ analyses). Effect sizes are reported for significant results.

The outcome variables of interest were the 10 questions (three replicated and seven new) on healthcare experiences relating to the diagnostic consultation (eg, ‘Overall, did you feel that the diagnostic consultation with this HCP was satisfactory?’). Responses were always binary (‘yes’/‘no’). First we explored differences on these outcomes using χ^2^ analyses; between the 1999 and 2013 samples (for the three replicated questions) and across the three subgroups within the 2013 survey sample (for all 10 questions). Then binary logistic regressions were used to assess factors associated with satisfactory healthcare experiences. Separate logistic regressions were carried out for each healthcare-experience question. Independent variables were the sociodemographic data, information on the respondent's eye condition and the main variable of interest—the survey groups (1999 vs 2013 samples for the three replicated questions, or the three subgroups of the 2013 survey sample for all 10 questions). Separate unadjusted logistic regressions explored the relationship between each predictor and the outcomes; then multivariable analyses with all predictors entered were conducted. The sample size fulfilled the requirement of >10 respondents for the lesser reported outcome event (ie, satisfaction or dissatisfaction) per predictor variable for multivariable logistic regression analyses (ie, events per predictor variable).^18^

Preliminary analyses indicated a general trend of increasing satisfaction with healthcare experiences over time. In order to assess the impact of the interventions, we controlled for this increasing trend by creating a continuous variable that ranked the respondents’ year of diagnosis (eg, the first year of diagnosis in the data set was 1980 and was coded as 1, and the last was 2013 and coded as 30). This variable was entered into the logistic regression analyses.

No problems with multicollinearity or linearity of the logit were observed.

Mann-Whitney tests explored differences in experiences with GPs between the 1999 and 2013 sample groups.

All analyses were conducted in SPSS V.21.0.

### Missing data and variables

In the 1999 survey, 709 respondents (58%) had missing data on whether they had wet or dry AMD. There were no questions with responses that correlated highly with this variable from the 1999 survey, so these data were not imputed, and this variable was not used as a predictor in the analyses comparing the 1999 and 2013 survey responses. Additionally, there was no question in the 1999 MDSQ on which HCP had first diagnosed the AMD, so this variable was not entered into analyses comparing the 1999 and 2013 samples.

There was a total of 289 respondents from the 1999 and 2013 surveys with missing data for age at first diagnosis (10.3%). The analyses reported here focus on AMD and so only those who were over the age of 50 at diagnosis and who had a diagnosis of AMD were included. Figure 1 explains how missing data were imputed to aid sample selection.

There were varying amounts of missing data on other independent variables, the greatest being for date of diagnosis (missing n=300, 11.4%). There were no suitable variables to allow imputation of these missing values. Analyses reported here comparing the 1999 and 2013 datasets are for those respondents with complete data on all independent variables included in the analysis: 1187 from the 1999-survey respondents and 1169 from the 2013 survey. Additional independent variables were entered into the 2013 survey-only analyses (some with missing data), and this left 1118 respondents. Item non-response was <5%. Sample size for each analysis will vary slightly depending on item non-response.

## Results

### Sample characteristics

Completed surveys were returned by 1545 respondents out of the 4000. Telephone completions by 26 gave a total sample size of 1571 for the 2013 sample (a 39% response rate). A further 267 uncompleted surveys were returned, with reasons for non-completion, giving a gross response rate of 46%. The most common reason given for non-completion was ‘old age’, followed by ‘ill health’. The MDSQ 1999 had been completed and returned by 1421 participants, including four telephone interviews (71% response rate).

For characteristics of the 1999 and 2013 survey samples, see table 1. Respondents to the 2013 survey were significantly older and less likely to be registered as either sight impaired (SI) or severely sight impaired (SSI) than the 1999 survey respondents. The two samples did not differ in gender, whether one or both eyes were affected by AMD or the time between diagnosis and survey completion.

**Table 1:** Respondent characteristics in the 1999 and 2013 survey groups

Variables	1999 sample (n=1187)	2013 sample (n=1169)†	Statistic, p value, effect size and n
Gender			
Male	368 (31.0%)	358 (30.6%)	χ^2^ (1)=0.04, p=0.84: n=2356
Female	819 (69.0%)	811 (69.4%)	
Age at survey completion (years)			
Mean (SD)	78.34 (7.10)	80.15 (7.99)	t (2313.83)=−5.79, p<0.001***: r=−0.02, n=2356
Median	78.66	81.00	
Registration status			
Not registered	469 (39.5%)	790 (67.6%)	χ^2^ (2)=206.53, p<0.001***: Cramer's V=0.30, n=2356
Registered sight impaired (SI)/partially sighted	379 (31.9%)	256 (21.9%)	
Registered severely sight impaired (SSI)/blind	339 (28.6%)	123 (10.5%)	
Number of eyes affected			
One eye	223 (18.8%)	226 (19.3%)	χ^2^ (1)=0.11, p=0.74: n=2356
Both eyes	964 (81.2%)	943 (80.7%)	
Years since diagnosis‡			
Mean (SD)	5.91 (4.92)	5.55 (3.77)	U=635, 839.00, z=0.30, p=0.77: n=2249
Median	5.00	5.00	

Values are frequencies (valid percentage %) unless otherwise stated.

*p<0.05; **p<0.01; ***p<0.001.

†The majority of the 2013 survey respondents self-reported their ethnicity as ‘white’ (99.5%). No question on ethnicity was included in the 1999 survey.

‡For information. Not included in further analyses.

Within the 2013 survey subgroups, respondents diagnosed after the 2009 RCOphth publication were younger, less likely to be registered, less likely to have both eyes affected by AMD and more likely to have been first diagnosed by an optometrist (table 2). Those diagnosed before the publication of the 2002 paper were more likely to have dry AMD and less likely to have wet AMD. Those diagnosed after the publication of the 2009 RCOphth guidelines were more likely to have wet AMD. There were no differences in gender balance within the 2013 subgroups.

**Table 2:** Respondent characteristics in the 2013 survey subgroups

Variables	Before MDSQ 1999 paper results publication (July 2002) (n=194)	Between 2002 and 2009 (n=448)	After RCOphth publication (February 2009) (n=476)	Statistic, p value: effect size
Gender				
Male	72 (37.1%)	135 (30.1%)	153 (32.1%)	χ^2^ (2)=3.02, p=0.22
Female	122 (62.9%)	313 (69.9%)	323 (67.9%)	
Age at survey completion (years)				
Mean (SD)	82.12 (7.26)	80.56 (7.68)	79.37 (8.16)	F (2, 1115)=8.86, p<0.001***: ω^2^=0.01†
Median	83.00	82.00	80.00	
Registration status				
Not registered	87 (44.8%)	269 (60.0%)	390 (81.9%)	χ^2^ (4)=108.67, p<0.001***: Cramer's V=0.22
Registered sight impaired (SI)/ partially sighted	62 (32.0%)	117 (26.1%)	67 (14.1%)	
Registered severely sight impaired (SSI)/blind	45 (23.2%)	62 (13.8%)	19 (4.0%)	
Number of eyes affected				
One eye	19 (9.8%)	61 (13.6%)	132 (27.7%)	χ^2^ (2)=42.76, p<0.001***: Cramer's V=0.20
Both eyes	175 (90.2%)	387 (86.4%)	344 (72.3%)	
Wet AMD only v dry AMD only v mixed wet and dry AMD				
Wet AMD only	43 (22.2%)	175 (39.1%)	195 (41.0%)	χ^2^ (4)=26.22, p<0.001***: Cramer's V=0.11
Dry AMD only	108 (55.7%)	176 (39.3%)	201 (42.2%)	
Wet and dry AMD	43 (22.2%)	97 (21.7%)	80 (16.8%)	
HCP who first diagnosed AMD				
Hospital eye specialist	131 (67.5%)	258 (57.6%)	230 (48.3%)	χ^2^ (2)=22.07, p<0.001***: Cramer's V=0.14
Optometrist	63 (32.5%)	190 (42.4%)	246 (51.7%)	

Values are frequencies (valid percentage %) unless otherwise stated.

*p<0.05, **p<0.01, ***p<0.001.

†ω^2^=0.01 represents a small effect size, 0.06 a medium effect size and 0.14 a large effect size.

AMD, age-related macular degeneration; HCP, healthcare professional; MDSQ, Macular Disease Society Questionnaire; RCOphth, Royal College of Ophthalmologists.

### Experiences in the diagnostic consultation: 1999 versus 2013 samples

Respondents from the 2013 survey were significantly more likely than the 1999 respondents to report feeling that the HCP who diagnosed their condition was interested in them as a person (71% compared with 47% see table 3). They were also more likely to report being given the name of their condition at diagnosis (91% vs 78%) and being generally more satisfied with the diagnostic consultation (76% vs 61%).

**Table 3:** Comparison of responses to questions on experiences within the diagnostic consultation across survey sample groups

	1999 survey sample	2013 survey sample	χ^2^, p value: effect size and n	2013 survey sample subgroups			χ^2^, p value: effect size and n
				Before MDSQ 1999 paper results publication (July 2002)	Between 2002 and 2009	After RCOphth guidelines publication (February 2009)	
Interested in you as a person?†	537 (46.9%)	805 (71.2%)	χ^2^ (1)=138.58, p<0.001***: φ=0.25, n=2276	126 (67.0%)	302 (70.1%)	351 (74.8%)	χ^2^ (2)=4.86, p=0.09: n=1088
Given the name of your condition?‡	906 (77.6%)	1045 (91.0%)	χ^2^ (1)=78.34, p<0.001***: φ=0.18, n=2315	165 (86.4%)	401 (91.1%)	435 (92.6%)	χ^2^ (2)=6.30, p=0.04*: Cramer's V=0.08, n=1101
Generally satisfied with diagnostic consultation?§	698 (61.0%)	856 (75.8%)	χ^2^ (1)=57.59, p<0.001***: φ=0.16, n=2273	129 (70.1%)	320 (73.7%)	382 (82.0%)	χ^2^ (2)=13.85, p=0.01*: Cramer's V=0.11, n=1084
Given written information?¶	–	–	–	34 (17.7%)	133 (30.4%)	193 (41.9%)	χ^2^ (2)=37.98, p<0.001***: Cramer's V=0.19, n=1090
Given appropriate support, help or advice?††	–	–	–	86 (45.7%)	229 (52.2%)	302 (64.1%)	χ^2^ (2)=23.25, p<0.001***: Cramer's V=0.15, n=1098
Information about the Macular Society?‡‡	–	–	–	28 (15.1%)	106 (24.0%)	150 (31.9%)	χ^2^ (2)=21.07, p<0.001***: Cramer's V=0.14, n=1097
Information on action if sudden deterioration in your vision?§§	–	–	–	80 (42.3%)	227 (51.4%)	262 (56.3%)	χ^2^ (2)=10.67, p=0.005**: Cramer's V=0.10, n=1096
Given information about likely progress of macular condition?¶¶	–	–	–	84 (43.5%)	203 (46.2%)	190 (40.6%)	χ^2^ (2)=2.94, p=0.23: n=1100
Other contacts for help and support?†††	–	–	–	29 (15.4%)	75 (17.1%)	90 (19.5%)	χ^2^ (2)=1.75, p=0.42: n=1088
Told about visual hallucinations?‡‡‡	–	–	–	26 (13.5%)	66 (15.4%)	80 (17.2%)	χ^2^ (2)=1.47, p=0.48: n=1085

Values are frequencies of ‘yes’ responses (valid percentage %) unless otherwise stated.

*p<0.05; **p<0.01; ***p<0.001.

†“Did you feel that this healthcare professional (who first diagnosed your macular condition), was interested in you as a person?” (Response ‘Yes’ or ‘No’ here and to all questions listed below).

‡“Were you given the name of your condition at the time of diagnosis?” (This question was included in the MDSQ 1999 but the responses were not reported in the 2002 paper).

§“Overall, did you feel that the diagnostic consultation with this healthcare professional was satisfactory?”

¶“Were you given any written information about your macular condition at the time of diagnosis?”

††“Do you feel you were given appropriate support, help or advice at the time of diagnosis?”

‡‡“Were you given information about the Macular Society (or the Macular Disease Society, as it was previously called) at the time of diagnosis?”

§§“Were you given any information around the time of diagnosis about what to do if you were to have a sudden deterioration in your vision?”

¶¶“Around the time of diagnosis, were you given information about the likely progress of your macular condition?”

†††“Were you given any other contacts for help and support at the time of diagnosis?”

‡‡‡“Were you told by a healthcare professional, around the time of diagnosis, of the possibility of experiencing visual hallucinations as a side effect of sight loss?”

MDSQ, Macular Disease Society Questionnaire; RCOphth, Royal College of Ophthalmologists.

Binary logistic regressions controlling for differences in sociodemographic and eye-related factors, confirmed that being a 2013-survey respondent was a significant predictor of satisfaction with these aspects of healthcare (see table 4 for odds ratios (ORs)).

**Table 4:** Predictors of healthcare experiences in comparable questions from the 1999 and 2013 surveys; unadjusted (univariable analyses) and multivariable analysis adjusting for all other predictors

Predictor	Interested in you as a person?† (n=2276)		Given name of condition?‡ (n=2315)		Overall satisfaction?§ (n=2273)	
	Unadjusted OR (95% CI)	Adjusted OR (95% CI)	Unadjusted OR (95% CI)	Adjusted OR (95% CI)	Unadjusted OR (95% CI)	Adjusted OR (95% CI)
**Sample** (1999 or 2013)						
1999	1.00	1.00	1.00	1.00	1.00	1.00
2013	2.80 (2.35 to 3.33)***	2.75 (2.28 to 3.31)***	2.92 (2.29 to 3.73)***	2.78 (2.14 to 3.61)***	2.00 (1.67 to 2.40)***	1.90 (1.56 to 2.31)***
**Gender**						
Male	1.00	1.00	1.00	1.00	1.00	1.00
Female	0.73 (0.61 to 0.87)**	0.71 (0.59 to 0.86)***	0.80 (0.63 to 1.03)	0.77 (0.60 to 1.00)*	0.68 (0.55 to 0.82)***	0.67 (0.55 to 0.82)***
**Age at survey completion¶**	1.02 (1.01 to 1.03)**	1.01 (1.00 to 1.02)	0.99 (0.97 to 1.00)	0.98 (0.97 to 1.00)*	1.03 (1.01 to 1.04)***	1.02 (1.01 to 1.04)***
**Registration status**						
*Contrast 1*						
Not registered	1.00	1.00	1.00	1.00	1.00	1.00
Registered as SI or SSI	0.50 (0.36 to 0.70)***	0.95 (0.64 to 1.42)	0.30 (0.19 to 0.47)***	0.60 (0.36 to 1.02)	0.62 (0.44 to 0.89)**	0.89 (0.59 to 1.35)
*Contrast 2*						
Registered SI	1.00	1.00	1.00	1.00	1.00	1.00
Registered as SSI	0.87 (0.68 to 1.10)	0.97 (0.75 to 1.25)	0.65 (0.48 to 0.88)**	0.72 (0.53 to 0.97)*	0.70 (0.55 to 0.91)**	0.74 (0.57 to 0.96)
**Number of eyes affected**						
One eye affected	1.00	1.00	1.00	1.00	1.00	1.00
Both eyes affected	0.78 (0.63 to 0.97)*	0.77 (0.60 to 0.97)*	0.78 (0.58 to 1.06)	0.92 (0.66 to 1.28)	0.78 (0.62 to 0.99)*	0.74 (0.58 to 0.96)*
**Adjusted model statistics**	–	χ^2^ (6)=160.91, p<0.001***	–	χ^2^ (6)=100.80, p<0.001***	–	χ^2^(6)=97.05, p<0.001***
**Adjusted model Nagelkerke's R^2^††**	–	0.09	–	0.07	–	0.06

*p<0.05, **p<0.01, ***p<0.001

†“Did you feel that this healthcare professional (who first diagnosed your macular condition), was interested in you as a person?” (Response was yes =1, no =0 for this and all questions below.)

‡“Were you given the name of your condition at the time of diagnosis?”

§“Overall, did you feel that the diagnostic consultation with this healthcare professional was satisfactory?”

¶In logistic regression, for continuous variables such as age at survey completion, an OR over 1 indicates increasing likelihood of the outcome as the predictor increases (ie, as age increases).

††Nagelkerke's R^2^ is a measure of model fit, where 0 indicates the predictors poorly predict the outcome and 1 is where the model predicts the outcome perfectly.

SI, sight impaired; SSI, severely sight impaired.

### Impact of 2002 publication of MDSQ results and 2009 RCOphth guidelines on healthcare experiences

Within the 2013 sample, a general pattern of increase in information provision and satisfaction with the diagnostic consultation was observed over the three time periods studied (prepublication in 2002, between 2002 and 2009, and post-2009 RCOphth guidelines) for all aspects of the consultation, apart from information provision on the likely progress of the macular condition (see table 3). Unadjusted logistic regressions using the ‘year rank’ variable found an increasing trend in information and support provision across time for six of the ten aspects of healthcare experiences (see table 5). Further unadjusted analyses using the 2013 survey subgroups found significant increases in the same six aspects of information and support provision after the 2002 paper publication compared with prepublication in 2002 (indicative of a combined effect of both interventions). However, there were significant improvements made after the 2009 RCOphth guidelines publication only for four aspects namely: information on the Macular Society, provision of written information, receiving appropriate support, help or advice at the time of diagnosis and overall satisfaction with the diagnostic consultation.

**Table 5:** Unadjusted ORs from binary logistic regressions investigating changes in information and support provision since the 2002 publication of the 1999 survey results and the 2009 RCOphth guidelines

	Interest as a person? OR (95% CI) n=1088	Overall satisfaction? OR (95% CI) n=1084	Name of condition? OR (95% CI) n=1101	Written information? OR (95% CI) n=1090	Info on deterioration? OR (95% CI) n=1096	Help and support? OR (95% CI) n=1098	Macular Society contact? OR (95% CI) n=1097	Other contacts? OR (95% CI) n=1088	Likely progress? OR (95% CI) n=1100	Hallucination? OR (95% CI) n=1085
**Sociodemographic**										
***Gender***										
Male	1.00	1.00	1.00	1.00	1.00	1.00	1.00	1.00	1.00	1.00
Female	0.73 (0.54 to 0.97)*	0.67 (0.49 to 0.91)*	0.99 (0.64 to 1.54)	0.79 (0.60 to 1.03)	0.78 (0.61 to 1.01)	0.68 (0.52 to 0.88)**	1.05 (0.79 to 1.40)	0.90 (0.65 to 1.25)	0.63 (0.49 to 0.81)***	0.98 (0.70 to 1.39)
***Age at survey completion‡***	1.01 (1.00 to 1.03)	1.03 (1.01 to 1.05)**	0.96 (0.93 to 0.98)**	0.98 (0.96 to 0.99)**	0.97 (0.95 to 0.98)***	1.01 (1.00 to 1.03)	0.99 (0.97 to 1.01)	1.01 (0.99 to 1.03)	1.00 (0.99 to 1.02)	1.03 (1.00 to 1.05)*
**Eye-related variables**										
***Number of eyes affected***										
One eye	1.00	1.00	1.00	1.00	1.00	1.00	1.00	1.00	1.00	1.00
Both eyes	0.58 (0.40 to 0.83)**	0.57 (0.38 to 0.85)**	0.87 (0.50 to 1.50)	0.60 (0.44 to 0.81)**	0.77 (0.57 to 1.04)	0.58 (0.42 to 0.80)**	0.55 (0.40 to 0.75)***	0.93 (0.63 to 1.38)	0.82 (0.61 to 1.11)	0.89 (0.60 to 1.33)
***Registration status***										
*Contrast 1*										
Not registered	1.00	1.00	1.00	1.00	1.00	1.00	1.00	1.00	1.00	1.00
Registered as SI or SSI	0.52 (0.30 to 0.92)*	0.65 (0.36 to 1.19)	0.16 (0.07 to 0.38)***	0.58 (0.33 to 1.02)	0.29 (0.17 to 0.49)***	0.31 (0.18 to 0.53)***	0.89 (0.49 to 1.62)	4.13 (2.16 to 7.91)***	0.82 (0.48 to 1.38)	2.92 (1.14 to 4.61)*
*Contrast 2*										
Registered as SI	1.00	1.00	1.00	1.00	1.00	1.00	1.00	1.00	1.00	1.00
Registered as SSI	1.11 (0.69 to 1.76)	0.63 (0.38 to 1.03)	0.48 (0.26 to 0.87)*	0.86 (0.53 to 1.40)	0.93 (0.60 to 1.44)	0.55 (0.35 to 0.85)**	1.15 (0.70 to 1.90)	1.11 (0.67 to 1.83)	1.05 (0.68 to 1.63)	0.82 (0.47 to 1.44)
***Wet or dry AMD***										
*Contrast 1*										
Wet AMD only and mixed wet and dry AMD	1.00	1.00	1.00	1.00	1.00	1.00	1.00	1.00	1.00	1.00
Dry AMD only	0.44 (0.26 to 0.75)**	0.48 (0.27 to 0.84)*	2.27 (0.94 to 5.51)	0.59 (0.35 to 1.01)	0.57 (0.35 to 0.92)*	0.29 (0.17 to 0.47)***	0.65 (0.37 to 1.15)	0.67 (0.35 to 1.28)	0.59 (0.36 to 0.97)*	0.44 (0.22 to 0.89)*
*Contrast 2*										
Wet AMD only	1.00	1.00	1.00	1.00	1.00	1.00	1.00	1.00	1.00	1.00
Mixed wet and dry AMD	0.67 (0.46 to 0.98)*	0.60 (0.40 to 0.90)*	1.17 (0.68 to 2.03)	0.76 (0.54 to 1.09)	0.82 (0.59 to 1.14)	0.62 (0.44 to 0.87)**	0.84 (0.58 to 1.22)	0.85 (0.56 to 1.31)	0.98 (0.71 to 1.37)	0.88 (0.57 to 1.36)
**Healthcare-related variables**										
***HCP who first diagnosed***										
Hospital eye specialist	1.00	1.00	1.00	1.00	1.00	1.00	1.00	1.00	1.00	n/a§
Optometrist	2.49 (1.88 to 3.30)***	2.40 (1.77 to 3.26)***	0.63 (0.42 to 0.96)*	0.73 (0.57 to 0.95)*	0.86 (0.68 to 1.10)	1.28 (1.01 to 1.63)*	0.94 (0.71 to 1.23)	0.91 (0.67 to 1.25)	1.11 (0.88 to 1.42)	n/a§
***Year of diagnosis (rank)‡***	1.02 (1.00 to 1.05)	1.03 (1.01 to 1.06)*	1.06 (1.03 to 1.10)**	1.10 (1.07 to 1.14)***	1.04 (1.02 to 1.07)***	1.07 (1.04 to 1.09)***	1.08 (1.05 to 1.11)***	1.02 (0.98 to 1.05)	0.99 (0.97 to 1.01)	1.02 (0.99 to 1.06)
***Survey time group***										
*Contrast 1*										
Before July 2002 publication of MDSQ 1999 results	1.00	1.00	1.00	1.00	1.00	1.00	1.00	1.00	1.00	1.00
After the 2002 publication (combined intervention effect)	1.58 (0.80 to 3.14)	2.24 (1.10 to 4.59)*	3.47 (1.33 to 9.02)*	7.31 (3.25 to 16.42)***	2.58 (1.36 to 4.91)**	2.84 (1.50 to 5.38)**	4.74 (1.99 to 11.29)***	1.37 (0.58 to 3.25)	0.96 (0.51 to 1.81)	1.41 (0.57 to 3.48)
*Contrast 2*										
Time between the 2002 paper publication and 2009 RCOphth guidelines publication	1.00	1.00	1.00	1.00	1.00	1.00	1.00	1.00	1.00	1.00
After RCOphth guidelines publication (February 2009)	1.29 (0.96 to 1.72)	1.63 (1.19 to 2.24)**	1.19 (0.74 to 1.92)	1.64 (1.25 to 2.16)***	1.22 (0.94 to 1.59)	1.63 (1.25 to 2.13)***	1.49 (1.11 to 1.99)**	1.19 (0.85 to 1.67)	0.80 (0.62 to 1.04)	1.16 (0.81 to 1.65)

*p<0.05; **p<0.01; ***p<0.001.

‡In logistic regression, for continuous variables such as age at survey completion, an OR over 1 indicates increasing likelihood of the outcome as the predictor increases (ie, as age increases).

§This question was not specific to the diagnostic consultation, but asked if they were told “around the time of diagnosis”.

AMD, age-related macular degeneration; HCP, healthcare professional; MDSQ, Macular Disease Society Questionnaire; RCOphth, Royal College of Ophthalmologists; SI, sight impaired; SSI, severely sight impaired.

Figure 2A–J shows the adjusted ORs for logistic regressions controlling for the impact of sociodemographic, eye-related and healthcare-related variables. In multivariable analyses, the trend for increasing satisfaction with healthcare experiences across time remained significant for the following three aspects of healthcare: provision of written information, information about the Macular Society and being given appropriate support, help or advice at the time of diagnosis. Once this trend was adjusted for, of the 10 aspects of care, only overall satisfaction with the diagnostic consultation significantly improved, and only after the 2009 RCOphth guidelines publication. There were no significant improvements associated with the 2002 publication of the MDSQ results.

**Figure 2:**
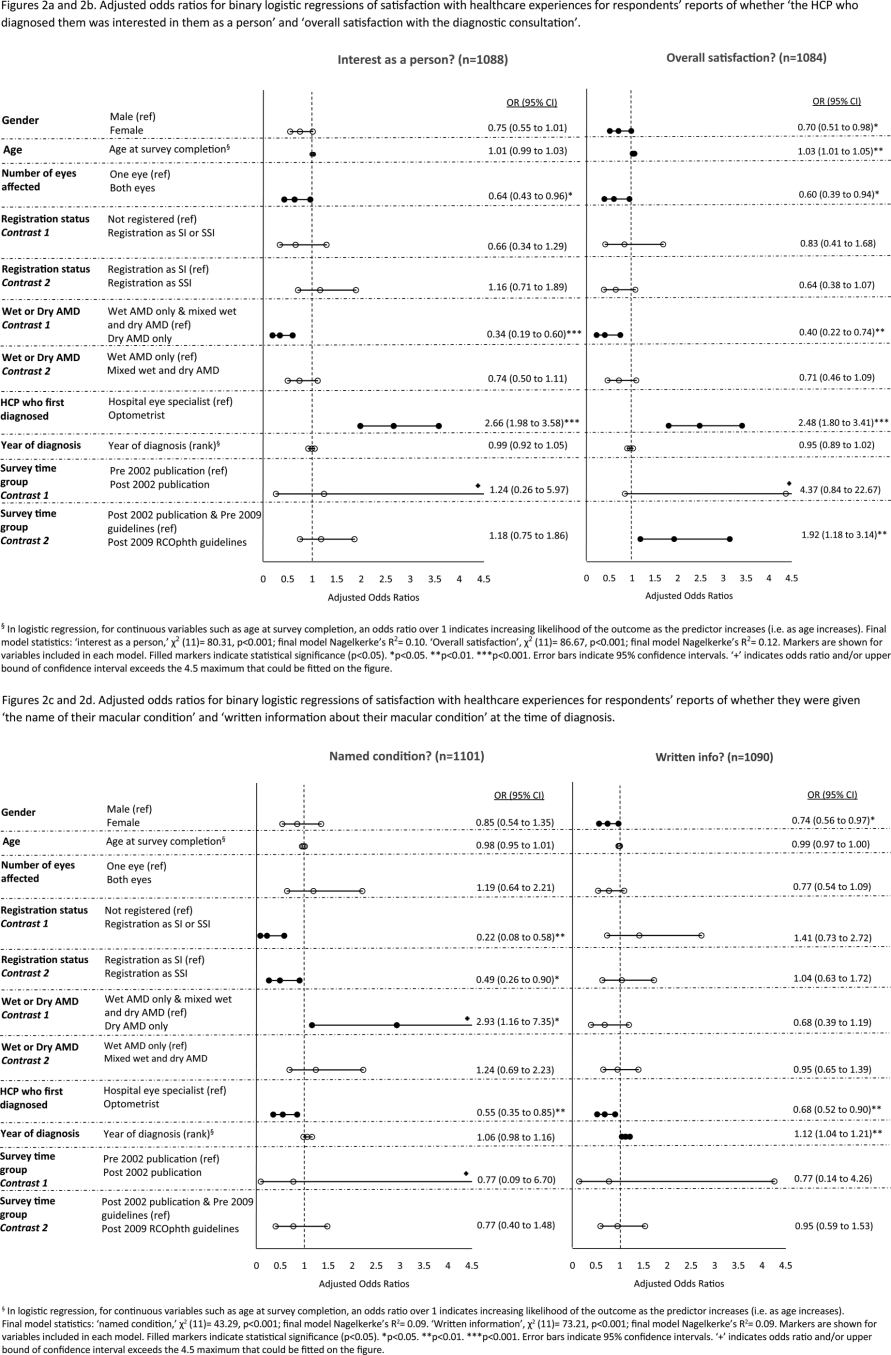
(A–J) Adjusted ORs for binary logistic regressions of satisfaction with healthcare experiences.

**Figure 2:**
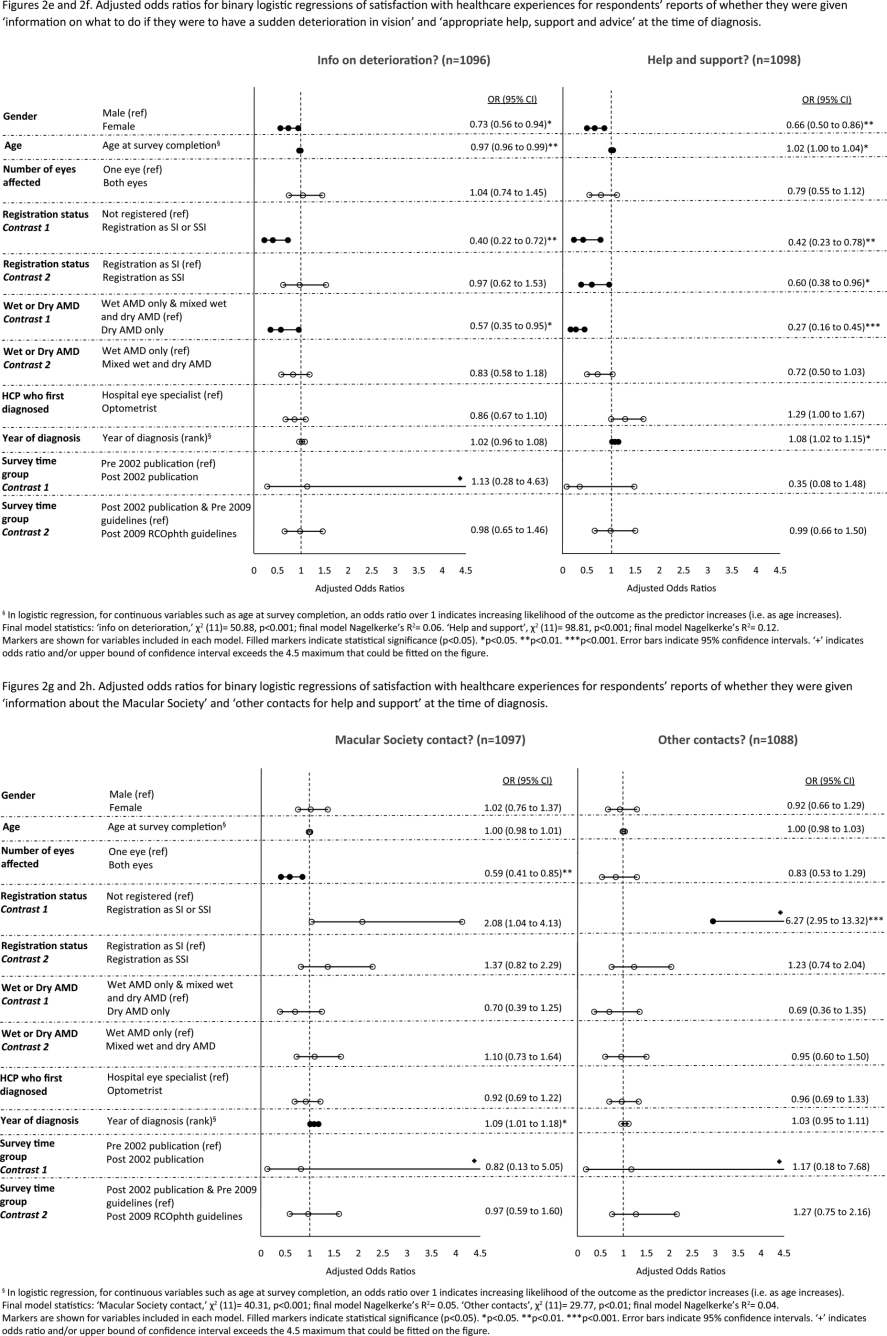
Continued

**Figure 2:**
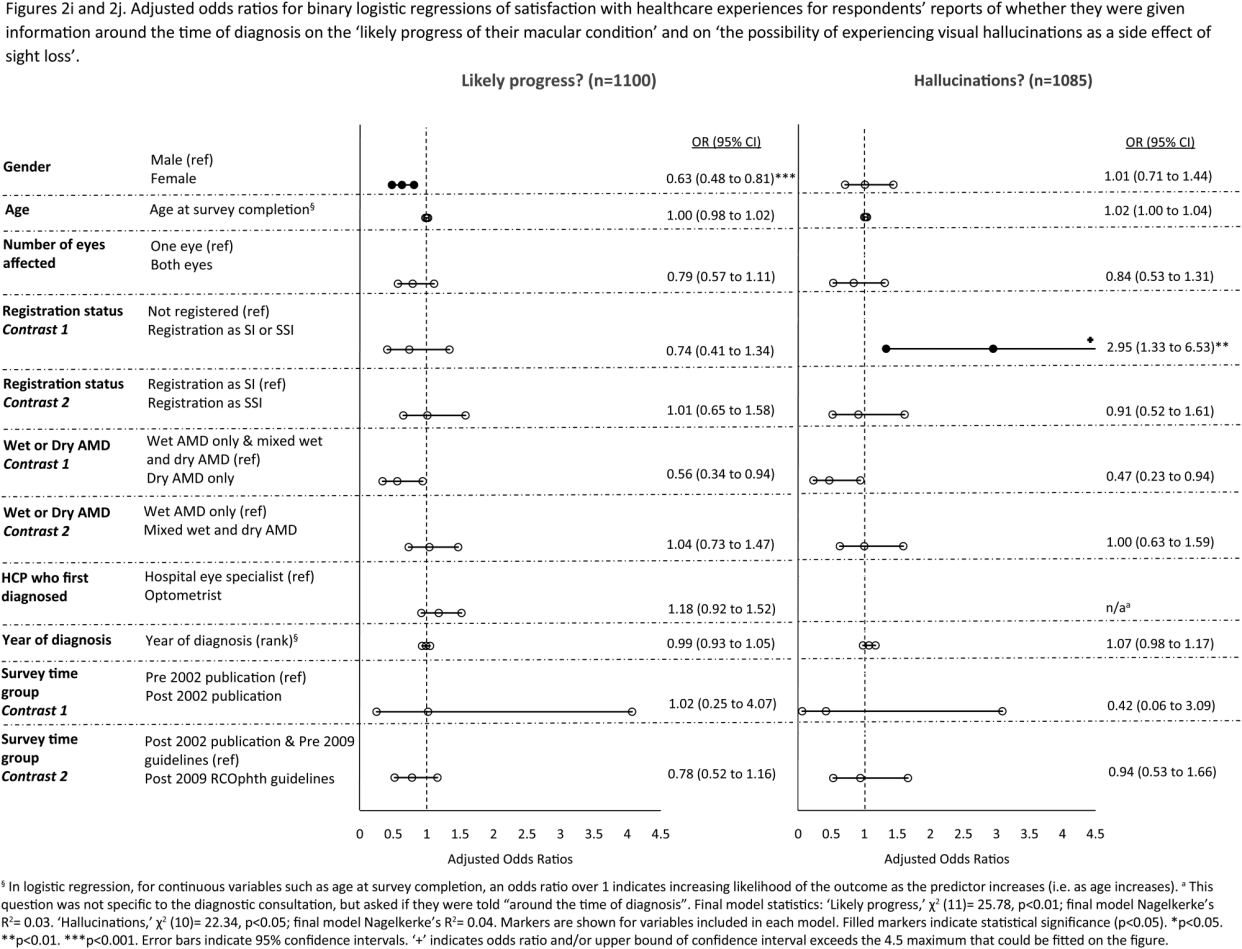
Continued

Women were less likely than men to report receiving information and support on five aspects of care (see figure 2A–J). Older respondents were more likely to report overall satisfaction with the diagnostic consultation and receiving appropriate help, support and advice at diagnosis, but were less likely than younger respondents to report receiving information on what to do if they have a sudden deterioration in their vision.

### Experiences with GPs around the time of diagnosis

**Figure 3:**
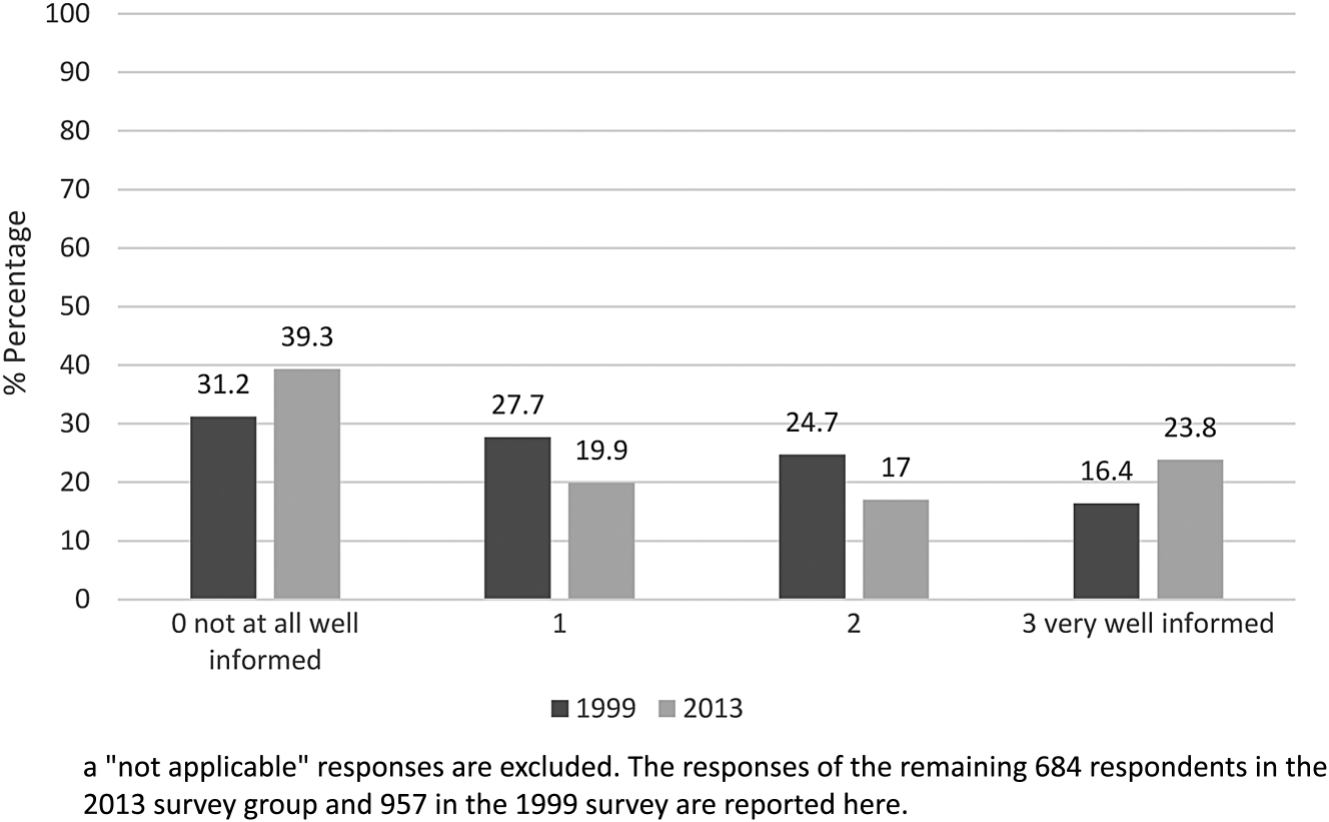
Comparison of the 1999 and 2013 survey responses to the question “Around the time you were first diagnosed with your macular condition, to what extent was your GP well-informed about your condition?”^a^

**Figure 4:**
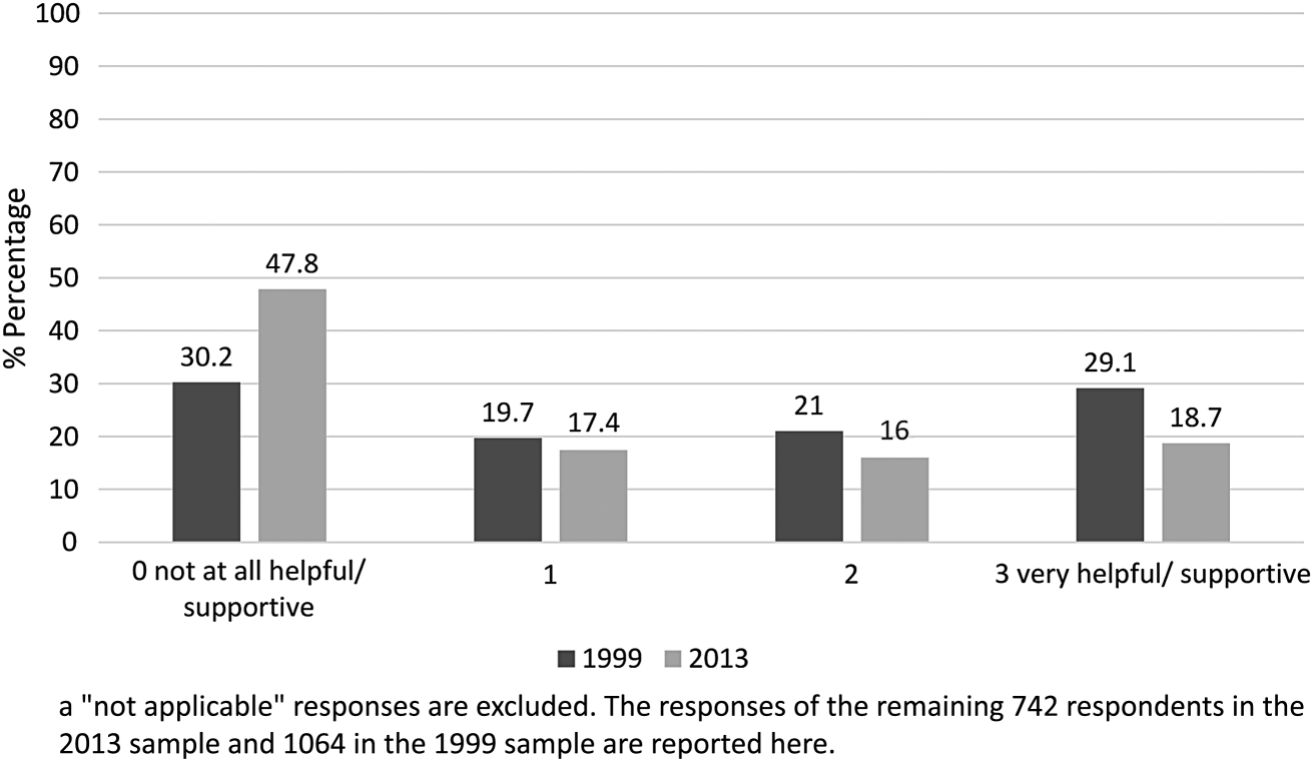
Comparison of the 1999 and 2013 survey responses to the question “To what extent has your GP been helpful and supportive about your macular condition?”^a^

Figures 3 and 4 show a comparison of the 1999 and 2013 survey responses on respondents’ views of GP knowledge about AMD, and help and support received from GPs in relation to their macular condition. In the 2013 survey, 163 respondents felt that their GP was ‘very well informed’ about their condition (23.8% of responses). However, many more said that their GP was ‘not at all well informed’ (n=269, 39.3%). Additionally, only 139 respondents (18.7%) reported that their GP was ‘very helpful and supportive’ about their AMD, and almost half of the survey respondents (47.8%) reported that their GP was ‘not at all helpful/supportive’ (n=355).

Mann-Whitney tests found no significant differences between the 1999 and 2013 samples in reported GP knowledge of AMD (U=321, 207.00, z=−0.67, p=0.50, n=1641) but there was a significant difference in reports of GP supportiveness. Respondents from the 2013 survey were more dissatisfied with the support provided by GPs (U=314, 740.00, z=−7.66, p<0.001, n=1806).

## Discussion

Patient experiences are an important indicator of quality of healthcare. This 2013 nationwide survey of people with AMD found significant improvements since a 1999 survey in patients being given the name of their macular condition at diagnosis, feeling that the HCP who first diagnosed them was interested in them as a person and overall satisfaction with the diagnostic consultation. Of two interventions that might have influenced this increase in satisfaction (the publication of the 1999 survey results and the 2009 publication of RCOphth recommendations for information and support provision to patients with AMD), only the latter was associated with significant improvements and this was only for overall satisfaction with the diagnostic consultation. Satisfaction with the three aspects of care measured in both surveys was high initially, reducing the scope for improvements which were nevertheless apparent over time.

The 2013 survey included seven newly designed questions based on RCOphth recommendations for information and support provision at diagnosis. Only three recommendations showed a significant trend of improvement over time (for written information on the macular condition, information on the Macular Society and for receiving appropriate help, support and advice at diagnosis). However, there were no additional improvements, over and above this general trend, following publication of the 2009 guidelines. The proportion of respondents reporting provision of this information and support remains low. Additionally, the 2013 survey respondents were more likely than the 1999 sample to report that their GP had not been helpful and supportive about their macular condition, and reported GP knowledge of AMD remains low.

This is the first large-scale survey to examine whether improvements in practice followed interventions designed to improve experiences of patients with AMD in the healthcare system. The survey is also unique in examining whether respondents felt they were receiving adequate support from their GPs for AMD. We have included several important sociodemographic, eye-related and healthcare factors linked to patients’ experiences in the multivariable analyses (table 4 and figure 2A–J). Of particular note is the association between registration as SI or SSI and lack of information provision at diagnosis on what patients need to do if they experience a sudden deterioration in vision. This finding suggests that lack of this information may cause subsequent sight loss sufficient to warrant registration. Respondents who were registered were also more likely to report not being given the name of their macular condition nor receiving appropriate help, support and advice in the diagnostic consultation. Respondents with dry AMD were less likely than those with wet AMD to be given information at diagnosis on what to do if they experience a sudden deterioration in vision. This is despite our current knowledge that if dry AMD turns to wet AMD, it is important that patients should seek help quickly as treatment is available that may prevent unnecessary sight loss.

Previous research has consistently found that older patients tend to be more satisfied with their healthcare experiences.^19–21^ There is some evidence also of women being less satisfied than men albeit a less consistent relationship.^20^ ^22^ ^23^ Our results show some similar findings and some differences. In this study, we were unable to establish whether differences in reported information and support provision were due to differences in patient characteristics (eg, expectations and recall) or differential treatment from HCPs providing less information and support to women than men, and less information but more help, support and advice to older people than younger people. Nevertheless, an awareness of these differences should prompt HCPs to check these particular ‘at risk’ groups have received and understood important information.

The response rate to the 2013 survey was low, but was not dissimilar to that of other surveys including the last nationwide General Practice Patient Survey (35.7% for 2015)^24^ and the Macular Society's survey the previous year.^17^ One might question the representativeness of our survey sample. Individuals may have joined the Macular Society because they had unsatisfactory experiences in their diagnostic consultations and sought information and support elsewhere. Conversely, this sample may have received information about the Macular Society in the diagnostic consultation more often than the general AMD population and thus be more satisfied. Members may have higher expectations of information and support than the general AMD population and be more likely to request information at diagnosis, if it is not offered. There has yet to be a large, geographically representative population study of AMD in the UK, so we are currently unable to estimate the representativeness of our sample. Additionally, no sociodemographic information was available on the non-responders to the survey to help estimate the representativeness of the final sample. As the most common reason stated by the ‘non-responders’ who returned paperwork was ‘old age’, followed by ‘ill health’, it was possible that those younger and in better general health were over-represented in our survey. However, the 2013 sample was on average older than the 1999 sample, and thus there seems not to be an over-representation of younger people in this sample. In comparison, non-responders to the 1999 survey cited ‘ill health’ or ‘visual impairment’ as the most common reasons for non-response. There were significantly fewer respondents registered as SI/SSI in the 2013 sample compared with the 1999 sample. Consequently, registration status was controlled for in the analyses in this report.

Respondents were asked to reflect on their experiences at diagnosis and this may be subject to recall bias. The use of survey methodology retrospectively to investigate patient experiences in consultations has been previously reported.^25^ Being diagnosed with a condition that could lead to sight loss is, in our experience of subsequent in-depth interviews with a subsample of respondents, a particularly memorable event for most people. The literature on autobiographical memory suggests that women recall more details than men.^26^ In this study, however, men were more likely to report receiving several aspects of information and support provision than women. This suggests that recall bias was not the main problem here but rather women may have received less information and support than men. The information that patients recall from their diagnostic consultation, even if asked years later, may still be relevant and important particularly for AMD where only a minority of patients are seen regularly—those receiving, or being monitored for treatment.

It is important to note that the results reported here demonstrate changes in information and support provision that occurred around the time of the interventions: we are not able to demonstrate direct cause and effect. The inclusion of the questions on experiences with GPs (who are unlikely to have read the RCOphth guidelines) could be seen to act as a control to test whether patient experiences would have improved across time regardless of the RCOphth guidelines. The lack of improvement in reports of experiences with GPs lends weight to the view that the RCOphth guidelines may well have had a positive influence on eye-care professionals which in turn may have improved overall patient satisfaction with the diagnostic consultation.

It will be important to investigate HCPs’ responses and explanations for the findings reported here. Perhaps the introduction of anti-VEGF injections for wet AMD has meant eye specialists are hard-pushed to find the time to provide adequate information and support in their diagnostic consultations? Perhaps eye-care professionals do not currently feel confident in providing the information recommended, for example, on the likely progress of macular conditions? Rates of diagnosis of AMD are expected to increase in the future, putting more pressure on eye clinics. High-quality written information for people with AMD, pertinent to the RCOphth guidelines, is provided to eye clinics free of charge by the Macular Society, but appears to be underused. (See https://www.macularsociety.org/resources for a list of resources.) This information might usefully be provided in community and primary care settings. Indeed our results indicate that 45% of respondents considered that it was their optometrist who first diagnosed their AMD, and many reported seeing their GP about their macular condition. It will be important to determine what obstacles are preventing the provision of written information to patients with AMD, and then take appropriate action to improve access to written material that is acceptable to patients and HCPs, and to monitor its impact. Previous research^27^ published in 2013 recommended a patient-centred approach to providing information about AMD. However, progress to date seems limited. Empathetic handling of the diagnosis, and support from HCPs is a priority^27^ and our results show that there is room for improvement.

Our findings suggest that, for people with AMD, information and support provision is low at diagnostic consultations with eye-care professionals and in GP consultations. There is a major opportunity here to improve patient experiences using an available patient information booklet at no expense to the health service. (See https://www.macularsociety.org/sites/default/files/resource/Macular%20Society%20Guide%20to%20AMD%202016_0.pdf). The expected benefits would be nationwide for the rising population of older people at risk of AMD.

## References

[1] Royal College of Ophthalmologists. Age-related macular degeneration: guidelines for management. London, England: Royal College of Ophthalmologists, 2009.

[2] Bourne RRA, Jonas JB, Flaxman SR Prevalence and causes of vision loss in high-income countries and in Eastern and Central Europe: 1990–2010. Br J Ophthalmol 2014;98:629–38. 10.1136/bjophthalmol-2013-304033 24665132

[3] Owen CG, Jarrar Z, Wormald R The estimated prevalence and incidence of late stage age related macular degeneration in the UK. Br J Ophthalmol 2012;96:752–6. 10.1136/bjophthalmol-2011-301109 22329913 PMC3329633

[4] Age-Related Eye Disease Study Research Group. A randomized, placebo-controlled, clinical trial of high-dose supplementation with vitamins C and E, beta carotene, and zinc for age-related macular degeneration and vision loss: AREDS report no. 8. Arch Ophthalmol 2001;119:1417–36. 11594942 10.1001/archopht.119.10.1417 PMC1462955

[5] Age-Related Eye Disease Study 2 Research Group. Lutein+zeaxanthin and omega-3 fatty acids for age-related macular degeneration: the Age-Related Eye Disease Study 2 (AREDS2) randomized clinical trial. JAMA 2013;309:2005–15. 10.1001/jama.2013.4997 23644932

[6] National Institute for Health and Care Excellence. *Ranibizumab and pegaptanib for the treatment of age-related macular degeneration* 2008 http://www.nice.org.uk/guidance/ta155

[7] Brown DM, Kaiser PK, Michels M Ranibizumab versus verteporfin for neovascular age-related macular degeneration. N Engl J Med 2006;355:1432–44. 10.1056/NEJMoa062655 17021319

[8] Brown DM, Michels M, Kaiser PK Ranibizumab versus verteporfin photodynamic therapy for neovascular age-related macular degeneration: Two-year results of the ANCHOR study. Ophthalmology 2009;116:57–65. 10.1016/j.ophtha.2008.10.018 19118696

[9] Rosenfeld PJ, Brown DM, Heier JS Ranibizumab for neovascular age-related macular degeneration. N Engl J Med 2006;355:1419–31. 10.1056/NEJMoa054481 17021318

[10] Martin DF, Maguire MG, Ying GS Ranibizumab and bevacizumab for neovascular age-related macular degeneration. N Engl J Med 2011;364:1897–908. 10.1056/NEJMoa1102673 21526923 PMC3157322

[11] National Institute for Health and Care Excellence. *Aflibercept solution for injection for treating wet age-related macular degeneration* 2013 http://www.nice.org.uk/guidance/ta294

[12] Amoaku WMK The Royal College of Ophthalmologists interim recommendations for the management of patients with age-related macular degeneration. Eye 2008;22:864–8.

[13] Chakravarthy U, Harding SP, Rogers CA Alternative treatments to inhibit VEGF in age-related choroidal neovascularisation: 2-year findings of the IVAN randomised controlled trial. Lancet 2013;382:1258–67. 10.1016/S0140-6736(13)61501-9 23870813

[14] Mitchell J, Bradley P, Anderson SJ Perceived quality of health care in macular disease: a survey of members of the Macular Disease Society. Br J Ophthalmol 2002;86:777–81. 12084749 10.1136/bjo.86.7.777 PMC1771190

[15] Royal College of Ophthalmologists. Age related macular degeneration: guidelines. London, England: Royal College of Ophthalmologists, 2000.

[16] Royal College of Ophthalmologists. Age-related macular degeneration: guidelines for management. London, England: Royal College of Ophthalmologists, 2013.

[17] Cox TM, ffytche DH Negative outcome Charles Bonnet Syndrome. Br J Ophthalmol 2014;98:1236–9. 10.1136/bjophthalmol-2014-304920 24825847 PMC4145458

[18] Peduzzi P, Concato J, Kemper E A simulation study of the number of events per variable in logistic regression analysis. J Clin Epidemiol 1996;49:1373–9. 8970487 10.1016/s0895-4356(96)00236-3

[19] Voutilainen A, Pitkäaho T, Vehviläinen-Julkunen K Meta-analysis: methodological confounders in measuring patient satisfaction. J Res Nurs 2015;20:698–714.

[20] Heje HN, Vedsted P, Sokolowski I Patient characteristics associated with differences in patients’ evaluation of their general practitioner. BMC Health Serv Res 2008;8:178 10.1186/1472-6963-8-178 18715502 PMC2533311

[21] Croker JE, Swancutt DR, Roberts MJ Factors affecting patients’ trust and confidence in GPs: evidence from the English national GP patient survey. BMJ Open 2013;3:e002762 10.1136/bmjopen-2013-002762 PMC3657663 23793686

[22] Elliott MN, Lehrman WG, Beckett MK Gender differences in patients’ perceptions of inpatient care. Health Serv Res 2012;47:1482–501. 10.1111/j.1475-6773.2012.01389.x 22375827 PMC3401395

[23] Nguyen Thi PL, Briançon S, Empereur F Factors determining inpatient satisfaction with care. Soc Sci Med 2002;54:493–504. 11848270 10.1016/s0277-9536(01)00045-4

[24] Ipsos MORI. GP Patient Survey National Summary Report. London: NHS England, 2016 http://gp-survey-production.s3.amazonaws.com/archive/2016/January/January+2016+National+Summary+Report.pdf (accessed 23 Jan 2016).

[25] Douglas G, Pavey S, Corcoran C Individual's recollections of their experiences in eye clinics and understanding of their eye condition: results from a survey of visually impaired people in Britain. Ophthal Physl Opt 2010;30:748–57. 10.1111/j.1475-1313.2010.00784.x 21205260

[26] Grysman A, Fivush R, Merrill NA The influence of gender and gender typicality on autobiographical memory across event types and age groups. Mem Cognit 2016;44:856–68. 10.3758/s13421-016-0610-2 27068433

[27] Burton AE, Shaw RL, Gibson JM ‘I'd like to know what causes it, you know, anything I've done?’ Are we meeting the information and support needs of patients with macular degeneration? A qualitative study. BMJ Open 2013;3:e003306 10.1136/bmjopen-2013-003306 PMC3822314 24202055

